# Conformations and Physicochemical Properties of Biological Ligands in Various Environments

**DOI:** 10.3390/ijms24119630

**Published:** 2023-06-01

**Authors:** Jean-Yves Le Questel

**Affiliations:** Laboratoire CEISAM, UMR CNRS 6230, Université de Nantes, F-44000 Nantes, France; jean-yves.lequestel@univ-nantes.fr

An accurate description of the conformational behavior of drug-like molecules is often a prerequisite for a comprehensive understanding of their behavior, in particular in the targeted receptor surroundings. Such studies are key in medicinal chemistry for the discovery of lead compounds and their optimization. The challenge of this quest is related to the conformational flexibility of drug-like molecules and the strong dependency on their conformational landscape as a function of the environment. In this context, a wide range of experimental and theoretical approaches, in many cases used in parallel, are available. From an experimental point of view, X-ray crystallography retains a prominent place since it provides not only the 3D structure of a compound but also a picture of its interaction potential. However, this picture is static, and the conformation adopted by the compound is heavily influenced by the crystal packing. On the other hand, NMR allows for structural information in solution to be obtained, but its time scale gives an averaged signal from the conformational ensemble, and theoretical studies must be carried out to obtain the corresponding individual relevant conformations. With respect to the theoretical side, quantum-chemistry-based methods, in particular density functional theory (DFT) calculations, provide accurate structural information but remain demanding in terms of time according to the size of the molecule and the conformational space under investigation. Force field methods remain attractive in this context and have the advantage of being able to probe the dynamics of the system (through molecular dynamics (MD) simulations), increasing the level of information available; however, they may suffer from a lack of suitable parameters for drug-like molecules. This research field therefore remains very active and also diverse, depending on the amount and level of experimental information available for the ligand considered.

A recent illustration of this research thematic can be seen in the study of Ziemnak et al. on halogen derivatives of 2-deoxy-d-glucose [1]. Through a combination of experimental methods (solid state and solution) and theoretical calculations using several quantum chemistry approaches, a comprehensive understanding of the structural features of halogen-substituted carbohydrates was acquired, as well as information on their intermolecular interactions, providing evidence of their distinct biological activity.

The design of new tools, based on the utilization of the information available in the world’s two major crystallographic databases, the Cambridge Structural Database (CSD) [2] and the Protein Data Bank (PDB) [3], fits perfectly into this research area. For example, the Torsion Library is a collection of torsion motifs encountered in low-molecular-weight ligands with angle distributions derived from crystallographic databases [4]. These features are of vital importance to drug design and may be a decisive factor in bioactivity. With them being based on experimental observations of a given motif in various intra and inter molecular environments, they encompass the corresponding conformational flexibility. Another example in this area is provided by CSD-CrossMiner, which allows for specific structural queries to be mined simultaneously in the CSD and the PDB [5]. As the number of experimental structures grows, the application potential of such tools should increase.

The investigations of integrin ligands’ conformational features carried out by Piarulli et al. exemplify this research field well [6]. For ligands with macrocycles, rigorous sampling of the corresponding conformations is required. In this study, conformational analysis of the ligands was realized experimentally through VT-NMR and NOESY experiments and computationally from Monte Carlo energy minimizations and MD simulations. These approaches allowed for the ligand conformational preferences necessary to investigate the ligand–receptor interactions to be obtained. 

The few examples mentioned above can be seen as illustrations of this research domain, which is productive in terms of publications and is rapidly evolving, on the basis of the design and implementation of new developments, both at the experimental and theoretical levels. In the context of this Special Issue dedicated to the field, six papers have been included, comprising two reviews and four research articles.

Based on a key property mentioned in the introduction of this editorial, related to the “bioactive” 3D conformation of a drug-like molecule, Bak provides a comprehensive review of 4D-QSAR [7]. It is apparent that 4D-QSAR has experienced a promising renewal of interest, with the rising power of graphics processing unit (GPU) clusters being notably at the heart of this resurgence.

The review by Novak et al. [8] addresses a key issue referred to in the first few lines of the present editorial. Indeed, understanding and characterizing the structure and interactions of macrolide antibiotics is crucial for the design of new antibiotics that are efficient against resistant pathogens. This issue is challenging since these compounds, owing to their macrocyclic structure, require specific tools for an adequate representation of their conformational landscape. Novak et al. provide a description of advanced methods, both experimental (phytochemical: X-ray, cryo-EM, NMR-based, and biochemical assays) and computational, mainly based on MD simulations, thus allowing this aim to be achieved. For the latter, the authors emphasize the need for proper force field parameters for macrolides. On the whole, this review illustrates in a comprehensive manner the requirement for various approaches that are able to probe the conformations of challenging systems in different environments, combining experiments and simulations to obtain a full and accurate picture of the structure and interactions of the parameters involved.

In the first article of this Special Issue, the authors use a combined experimental (fluorimetric measurements) and molecular modeling (semi-empirical calculations) approach to investigate the binding of the anti-metabolite drug methotrexate with a cavitand derivative [9]. The experiments allow for the determination of the thermodynamic parameters of the host–guest interaction, while molecular modeling enables the structure of the various possible complexes to be brought to light.

The work of Cerrón-Carrasco is exclusively based on DFT calculations and knowledge of the key structural and physicochemical properties of anti-cancer drugs. The work shows the power of these methods to generate a series of bifunctional prodrugs aimed at reducing the toxicity of these compounds through an increase in the oxidation state of the metallic center (Pt(II) to Pt(IV)) and its combination with recent FDA-approved anti-cancer drugs [10]. The above strategy is encouraging since a promising oncology drug emerged from this study.

The study by Korlyukov et al. focuses on the conformational properties of imatinib-containing compounds through the use of a combined strategy based on crystal structure (CSD and PDB) analyses and DFT calculations [11]. Imatinib is one of the most used therapeutic agents against leukemia, and its flexibility as well as an important number of specific and non-specific interaction sites make the characterization of its structural and interaction properties challenging. The strategy employed sheds light on the contribution of the various interactions (H-bond and π–π) in the behavior of the compound and their relationship with the possible conformations.

In the final article included in this Special Issue, a new ligand for the 5-HT1A and 5-HT7 receptors, an arylpiperazine salicylamide derivative with an inflexible spacer, is thoroughly investigated using experimental physicochemical methods at the solid state (X-ray crystallography and NMR) and periodic DFT calculations [12]. These methods first enable the characterization of ligand conformation in different solvates, information relevant to the synthesis of new active pharmaceutical ingredients. Secondly, the characteristic features of the interactions at work (H-bonds: NH–O and CH–O and aromatic bonds: CH–π and π–π) are determined as well as their contribution to the structure of the different solvates.

The above papers are examples of the applications of a broad range of techniques, both experimental and theoretical, that can be used alone but most often in combination, to provide a comprehensive understanding of the structure and interaction potential of drug-like molecules in different environments. The subject remains of distinct importance and, therefore, a very active research field, since, in particular, understanding of the molecular recognition process is at the heart of drug discovery.
